# Drug Repurposing Targeting *Pseudomonas aeruginosa* MvfR Using Docking, Virtual Screening, Molecular Dynamics, and Free-Energy Calculations

**DOI:** 10.3390/antibiotics11020185

**Published:** 2022-01-31

**Authors:** Tatiana F. Vieira, Rita P. Magalhães, Manuel Simões, Sérgio F. Sousa

**Affiliations:** 1 UCIBIO/REQUIMTE, BioSIM, Departamento de Medicina, Faculdade de Medicina da Universidade do Porto, Alameda Prof. Hernâni Monteiro, 4200-319 Porto, Portugal; tatianafvieira@gmail.com (T.F.V.); ritaprata1@hotmail.com (R.P.M.); 2 Associate Laboratory i4HB, Institute for Health and Bioeconomy, Faculdade de Medicina, Universidade do Porto, 4200-319 Porto, Portugal; 3 LEPABE Laboratory for Process Engineering, Environment, Biotechnology and Energy, Faculty of Engineering, University of Porto, Rua Dr. Roberto Frias, s/n, 4200-465 Porto, Portugal; mvs@fe.up.pt

**Keywords:** drug repurposing, *Pseudomonas aeruginosa*, computer-aided drug design (CADD), biofilms, quorum sensing

## Abstract

*Pseudomonas aeruginosa* is an opportunistic Gram-negative bacterium responsible for acute and chronic infections in planktonic state or in biofilms. The sessile structures are known to confer physical stability, increase virulence, and work as a protective armor against antimicrobial compounds. *P. aeruginosa* can control the expression of genes, population density, and biofilm formation through a process called quorum sensing (QS), a rather complex and hierarchical system of communication. A recent strategy to try and overcome bacterial resistance is to target QS proteins. In this study, a combined multi-level computational approach was applied to find possible inhibitors against *P. aeruginosa* QS regulator protein MvfR, also known as PqsR, using a database of approved FDA drugs, as a repurposing strategy. Fifteen compounds were identified as highly promising putative MvfR inhibitors. On those 15 MvfR ligand complexes, molecular dynamic simulations and MM/GBSA free-energy calculations were performed to confirm the docking predictions and elucidate on the mode of interaction. Ultimately, the five compounds that presented better binding free energies of association than the reference molecules (a known antagonist, M64 and a natural inducer, 2-nonyl-4-hydroxyquinoline) were highlighted as very promising MvfR inhibitors.

## 1. Introduction

*Pseudomonas aeruginosa* is a highly adaptable Gram-negative bacterium, responsible for acute and chronic infections that rapidly evolve to multi-drug resistance to antibiotics, and is becoming extremely difficult to eradicate, leading to high morbidity and mortality rates [1,2]. It can be found in planktonic state or as aggregates, called biofilms. This association of microorganisms is the ultimate way of protection to adverse conditions [3] and involves a self-produced matrix of extracellular polymeric substances that confers stability and protection from external stress conditions [4,5,6,7].

*P. aeruginosa* has a mechanism of intracellular communication that controls the population density, expression of genes, and biofilm formation called quorum sensing (QS). It involves the production, detection, and response to extracellular signaling molecules, called autoinducers (AIs) [2,8,9]. QS in *P. aeruginosa* is rather complex and hierarchical. Until now, for *P. aeruginosa*, there are four types of signaling systems described. Two are based on acyl homoserine lactones (LasR and RhlR): one that uses quinolone as signaling molecule (PQS) and one whose mechanism and targets are still unknown (IQS) [10,11]. All these systems communicate with each other to control the direct and indirect expression of several virulence genes [12]. Details on the intricate web of interactions between these systems have already been defined extensively by several other groups [1,13,14,15,16]. 

A recent strategy to try to overcome bacterial resistance is to target QS proteins, inhibiting their activity. In this way, the bacterial virulence is eliminated, but the bacteria do not die, leading to lower acquired and adaptive resistance [1,17,18]. Targeting the PQS system may be advantageous because it is specific to *P. aeruginosa*. By not interfering with other QS systems, such as las and rhl, there is minimization of potential side effects, as bacteria that are essential to human health are not affected [19].

*Pseudomonas aeruginosa* QS regulator (PqsR), also called a multiple virulence factor regulator (MvfR), is a LysR-like transcriptional regulator protein (LTTRs) [20] common in many species of bacteria. It has a highly conserved DNA binding domain and a poorly conserved ligand binding domain, also called a co-inducer or a ligand-binding domain (CBD). The natural AIs are: 2-Heptyl-3-hydroxy-4(1*H*)-quinolone (also called the Pseudomonas Quinolone Signal, PQS) and, its precursor, 2-heptyl-4-hydroxyquinoline (HHQ). The synthesis and regulation of these AIs are controlled by the pqsABCDE operon which, in turn, is regulated by MvfR, creating an auto-regulatory loop. The expression of MvfR, on the other hand, is controlled by LasR [1,14,20,21,22].

This protein is not only an important target due to the direct and indirect activation of a variety of virulence genes, but it has been proven that it is also responsible for the formation and development of biofilms as well as antibiotic-tolerant persister (AT/P) cells [23]. Thus, this is an excellent target to control and treat *P. aeruginosa* infections. 

**Figure 1:**
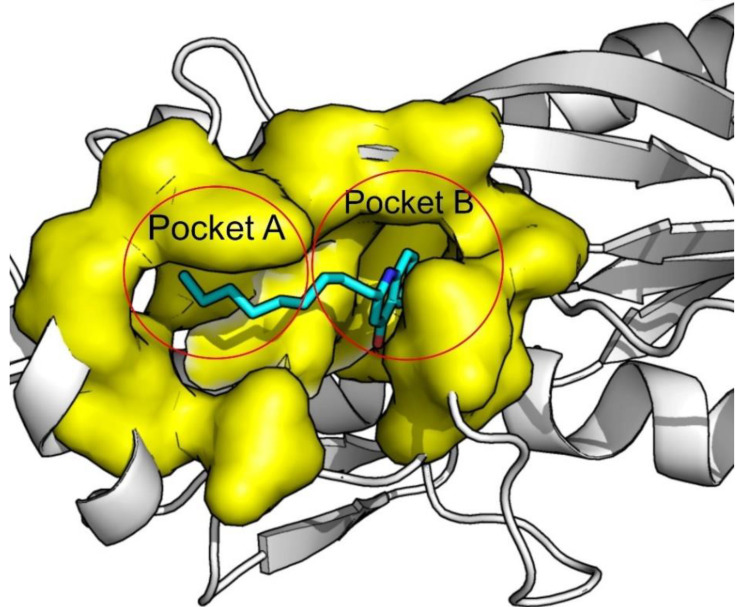
MvfR pocket A and pocket B depiction. Protein bound to its natural inducer NNQ. (PDB:4JVI).

The binding pocket of PqsR/MvfR has two subdomains, defined as Pocked A and Pocket B. The bicyclic ring of the natural autoinducer fits perfectly into pocket B and the aliphatic chains interact with residues on pocket A, as represented in Figure 1. It is a mainly hydrophobic pocket, which is one of the reasons why it is quite a challenging and high-interest target [24,25].

A review paper [26] was published describing the recent advances in the development of MvfR antagonists, leading the way to the rational design of new and specific drugs to treat *P. aeruginosa* infections. Analyzing all the compounds described, it is clear that there are common features and it is even possible to design a pharmacophore model [26]. To sum up, so far, the classes of molecules that have been tested and present inhibitory activity against MvfR are quinazolinones, quinolinones, benzamide–benzimidazoles, and hydroxybenzamides [20,23,24,27,28,29,30]. However, it is still unknown if these compounds will work on their own or if it is still necessary to use antibiotics, as there are still no clinical trials being made on this subject.

In this work, docking and virtual screening experiments were applied to discover new inhibitors for PqsR/MvfR using the ZINC FDA-approved database as a starting point. De novo synthesis is a very time-consuming and expensive process, and the search for new antibiotics has led to disappointing results with the main restricting factor being the lack of translation of in vitro activity in the live bacterial cells [31]. Drug repurposing for antimicrobial purposes aims to generate new clinical uses for already-established drugs with possible antibiotic effects or potentiate it when used in combination with an antibiotic [31,32].

## 2. Results and Discussion 

The results presented in Table 1 show that the scoring functions (SFs) that can more effectively reproduce the binding poses of the crystallographic ligands in MvfR are GOLD’s CHEMPLP and ASP, with an average RMSD of 1.78 and 1.81 Å, respectively. ChemScore and VINA also gave reasonable results with average RMSD values of 3.00 Å and 3.57 Å, respectively. The worst results were obtained with LeDock and GoldScore with average RMSD values of 4.51 Å and 3.27 Å. Within individual scoring values, there were also significant variations among the different PDB–ligand combinations, arising from the diversity of molecules considered. QZN, NNQ, HLH, QAE, and Q25 have long aliphatic tails that are very flexible and hydrophobic, and many of the scoring functions could not reproduce the exact crystallographic pose and, hence, the high RMSD values. M64 is less flexible and bulkier, and most scoring functions were able to reproduce the original pose. Only ChemScore failed this test with a RMSD of 3.48 Å.

The same tendency is seen when we analyze the remaining data. It is more challenging for all the scoring functions to reproduce the crystallographic pose in ligands with long, aliphatic chains.

**Figure 2:**
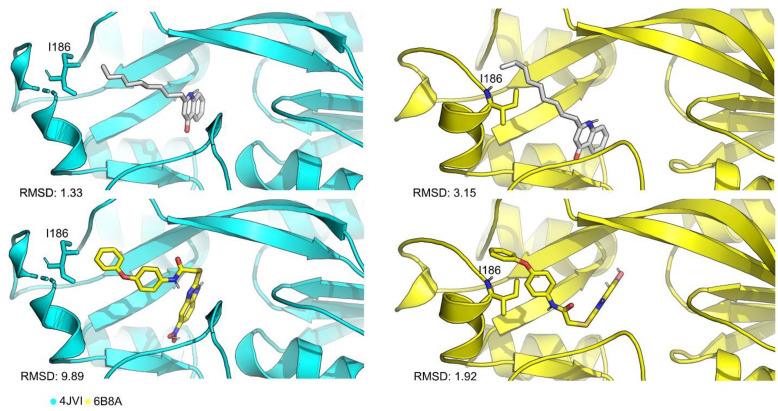
Cartoon representation of the active site residues for 4JVI (cyan), 6B8A (yellow), NNQ ligand (white) and M64 (yellow). The main difference in these three structures is the position of residue Ile186 that in 6B8A structure tends to close the binding site, accommodating bulkier ligands.

Upon further analysis, it was clear that the different SF showed a clear tendency in RMSD values and there were two targets that stood out: 4JVI and 6B8A. In these cases, the cross-docking scores (Supplementary Table S1) are consistently higher than any other target. 4JVI seems to accommodate ligands with aliphatic chains better, while 6B8A showed good scores for different types of ligand structures. The main difference between the two structures lies in the position of residue Ile186, as seen on Figure 2—a feature that can be important to accommodate a variety of ligands in a virtual screening (VS) run. At the cross-docking stage with structure 4JVI, the SFs were not able to dock M64 in the same pose as the crystallographic due to the position of Ile186, evidenced by the RMSD values.

Based on the results presented, two main structures were chosen (PDB codes: 4JVI and 6B8A) to continue the experiments, for the validation of the virtual screening (VS) protocol. Furthermore, at this stage, LeDock and GoldScore SFs were eliminated due to poor RMSD results.

**Figure 3:**
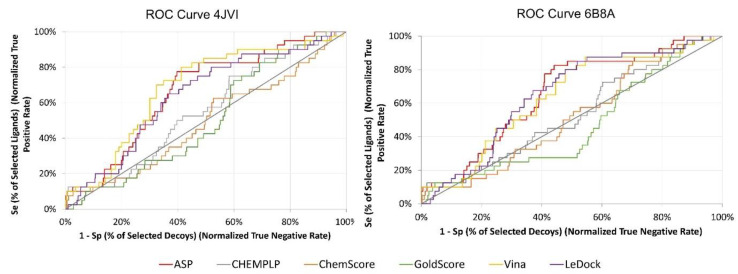
ROC curves for 4JVI and 6B8A.

Docking of the actives vs decoys dataset (2290 compounds) was performed against only the two PDB structures selected previously (4JVI and 6B8A). At the end of the calculations, each compound was ranked according to their binding score and the ROC curves were plotted (Figure 3). The curves show similar results for both targets, with GoldScore and LeDock being the worst at discriminating between binders and non-binders early on. 

Table 2 summarizes the results obtained for these two targets for all scoring functions tested. Vina, CHEMPLP, ChemScore, and ASP provided good discriminatory ability between binders and non-binders, with ChemScore giving the lowest enrichment factor (EF) 1% for both targets. For 4JVI, Vina, CHEMPLP, and ASP provided the best EF 1% but then presented TG below 0.25. For 6B8A, Vina and ASP consistently provided the best results across most of the metrics studied. ASP exhibited an EF of 10%, an AUC above 0.5, and a TG of 0.25, indicating good performance and reproducibility of the protocol. Ultimately, ASP was the SF chosen to proceed to the VS of the FDA-approved database.

After performing the virtual screening protocol for the ZINC FDA-approved database, the molecules present in the top 1% were analyzed in detail, corresponding to 32 compounds. The top 15 molecules identified in the VS stage are listed in Table 3, along with their respective structure and docking score. A brief description of the pharmaceutical use of each compound is also provided.

Mellini and co-workers performed a similar VS study using a library of 1467 FDA-approved drugs against MvfR [25]. The five top hits were selected (Conivaptan, Ergotamine, Eltrombopag, Pimozide, and Dutasteride) and tested experimentally in in vitro assays. Only pimozide presented inhibitory activity toward MvfR. Eltrombopag was also one of the compounds present in the top 1% of our VS results (rank position nº 19) but, since this, a different protocol and a different SF were used; thus, it is only logical that different hits were obtained. Among the collection of compounds selected, the protocol was able to place two antibiotics in the top 15 results which reinforces the virtual screening methodology even further.

The subsequent stage of the study involved performing the 100 ns of MD simulations for each complex with 6B8A, followed by MM/GBSA calculations. As reference, a MD simulation of NNQ in complex with 6B8A was also performed. NNQ is one of the natural agonists of MvfR. The first antagonist with activity against MvfR and M64 was also used as reference.

The stability of the complexes was accessed by RMSD calculations for the Cα of the protein and ligands as well (supporting information for more detail). As presented in Table 4, all the MvfR–ligand complexes displayed low protein and ligand RMSD values throughout the simulations meaning that they are indeed stable.

**Figure 4:**
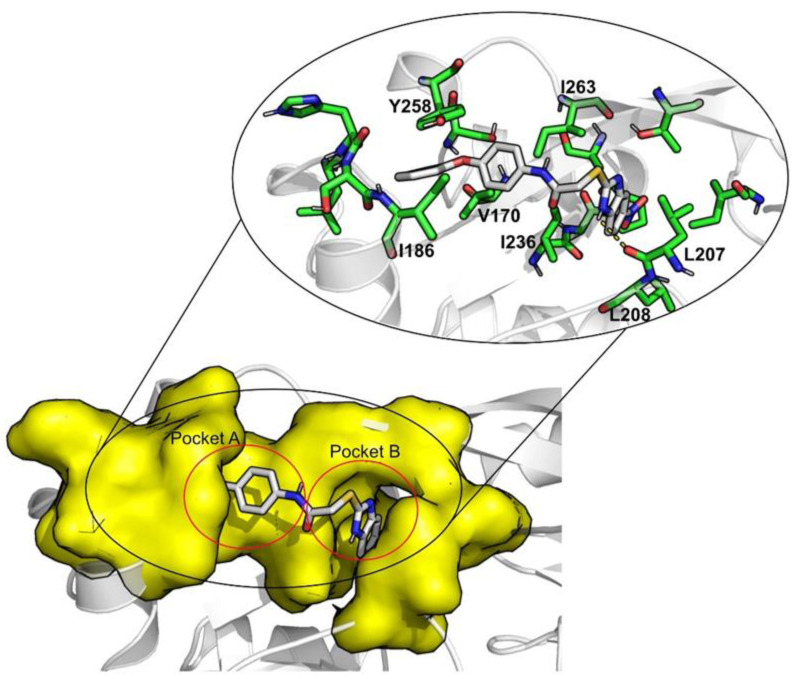
6B8A–M64 interaction map.

Smaller average SASA values and a higher percentage of average buried ligand area indicate that the ligand is less exposed to the solvent, more shielded by the pocket, also suggesting greater stability. Of all the compounds simulated, isavuconazonium and methotrexate are these more exposed to the solvent (522.5 ± 47.4 Å^2^; 42.7 ± 0.1% and 312.6 ± 69.2 Å^2^; 53.8 ± 0.1%, respectively). The reference compound M64 exhibits the lowest SASA and higher percentage of potential ligand SASA buried (90.4 ± 19.3 Å^2^; 86.3 ± 0.03%), mainly because of the π–π interaction with Tyr258 and the ring portion of M64. The position of this residue and the position of Ile186 seem to be important for the “closing” or “opening” of pocket A, as seen on Figure 4.

Of the 15 FDA compounds studied, eight presented better binding free energies than the reference, M64 (−39.0 ± 0.1 kcal/mol). These were venetoclax (−70.1 ± 0.3 kcal/mol), indocyanine green (−58.6 ± 0.3 kcal/mol), nilotinib (−48.1 ± 0.2 kcal/mol), cabozantinib (−44.6 ± 0.2 kcal/mol), montelukast (−43.2 ± 0.2 kcal/mol), cefoperazone (−42.4 ± 0.4 kcal/mol), valrubicin (−41.1 ± 0.2 kcal/mol), and lomitapide (−40.6 ± 0.3 kcal/mol). The compounds highlighted as of potential therapeutic interest to target QS consider the SASA, % of buried ligand, and binding free-energy values, narrowing the selection to the top five (venetoclax, indocyanine green, nilotinib, cabozantinib, and montelukast).

**Figure 5:**
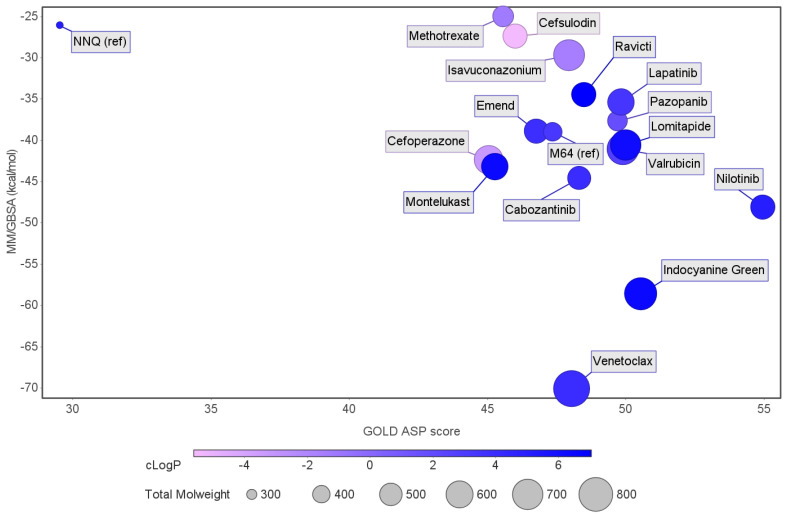
Graph comparing the free-energy calculation results using MM/GBSA with the GOLD ASP scores. The reference ligands are NNQ, one of MvfR auto-inducer molecules, and M64, an antagonist. Blue-colored ligands indicate a higher LogP than those colored in pink. The size of the marker is a representation of the molecular weight.

Figure 5 compares the results from the MM/GBSA calculations and the GOLD ASP scores. The graph indicates that all the compounds selected present a higher affinity toward MvfR than the reference ligand, with venetoclax (−70.1 ± 5.7 kcal/mol for MM/GBSA), showing the highest affinity of all the molecules in the set. Isavuconazonium (−25.0 ± 0.1 kcal/mol), methotrexate (−22.8 ± 0.3 kcal/mol), and cafsulodin (−27.4 ± 0.3 kcal/mol) were the compounds with the lowest binding affinity, as previously mentioned. There is a clear tendency in terms of LogP values, with the most promising drugs showing higher LogP values. This can be an important aspect to consider because these compounds need to cross the bacterial cell wall to reach their specific target. The molecular weight of all the molecules selected is above 500 g/mol.

**Figure 6:**
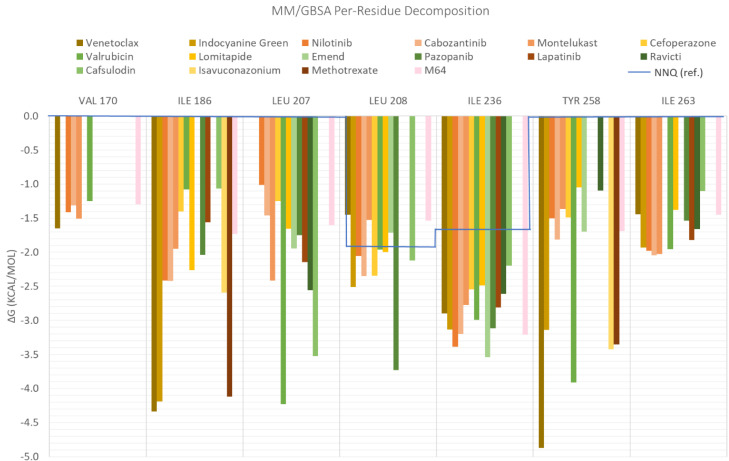
Per-residue decomposition of the free-energy calculations using MM/GBSA for the fifteenth selected FDA compounds.

When the free-energy values obtained using MM/GBSA were decomposed, the contribution of each residue was analyzed. There are three main residues that contribute to an increased affinity in all MvfR–ligand complexes: Ile186, Ile236, and Ile263. The contributions of Val170, Leu207, Leu208, and Tyr258 does not exist for all the complexes, but their presence benefits the interaction for nilotinib, valrubicin, venetoclax, isavuconazonium, montelukast, and cefoperazone (Figure 6). Lapatinib also interacts strongly with Val211 and cefoperazone with Arg209 (data not shown in Figure 6).

When comparing these residues with the main residues interacting with NNQ (blue line), there was an increase in the number of interacting residues (in the NNQ case the interacting residues are only Leu208 and Ile236). This means that the association between these 15 selected FDA molecules is stronger than the interaction with its autoinducer. This might be explained by the fact that NNQs has a big aliphatic chain and the interactions in the binding pocket are mainly due to hydrophobic effects.

**Figure 7:**
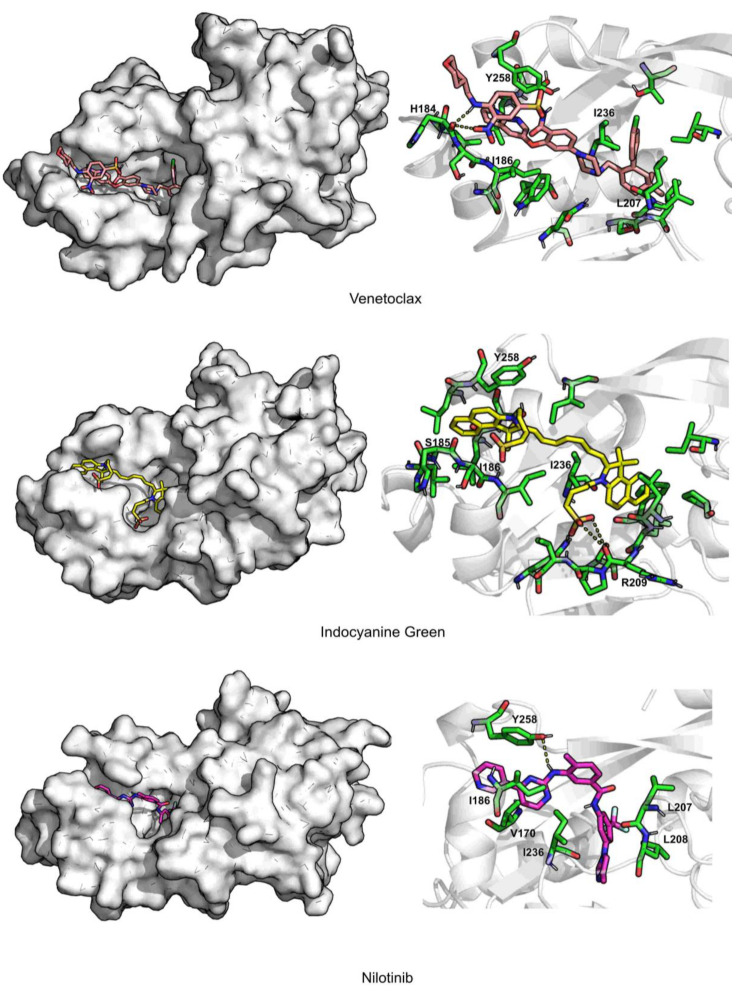
Interaction maps for venetoclax, indocyanine green, and nilotinib.

**Figure 8:**
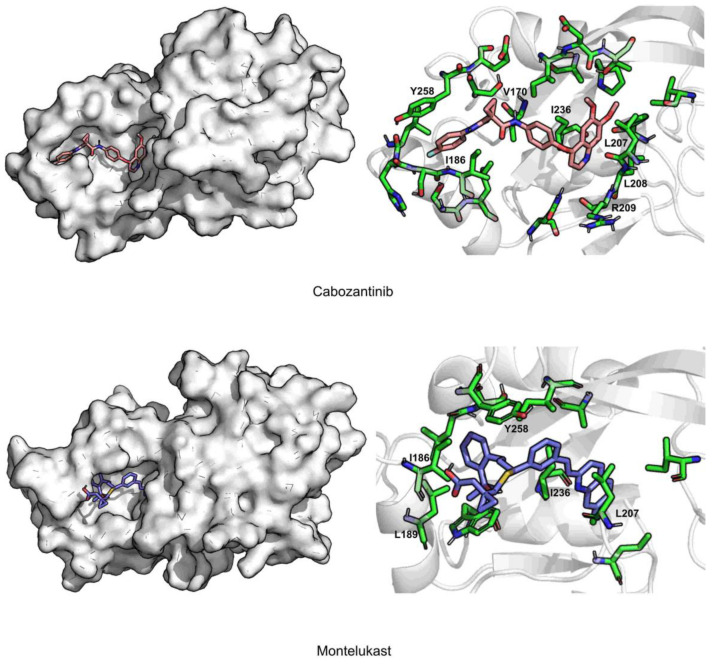
Interaction maps of cabozantinib and montelukast.

In Figure 7 and Figure 8, the interaction maps of the five selected FDA-approved compounds are depicted. Venetoclax is stabilized through hydrophobic interactions with residues Leu207, Tyr258, and Ile236. It can form hydrogen bond interactions with His184. It is a big molecule and occupies the total area of pocket B and pocket A, and also induces a shift in the position of Tyr258, opening pocket A even more.

Indocyanine green is mainly stabilized through hydrophobic interactions with residues Ile186, Leu207, and Ile236. It can form π–π interactions with Tyr258 and the oxygen atoms of the sulfate group form a salt bridge with Arg209. Indocyanine green causes a shift in the position of Tyr258 and Ile186, inducing the opening of pocket A.

Nilotinib is stabilized by hydrophobic interactions with residues Val170, Ile186, Leu208, Ile236, and Tyr258. Tyr258 also forms a hydrogen bond with nilotinib. It also induces a shift in the position of Tyr258 and Ile186, causing an opening of the binding pocket.

Cabozantinib is stabilized by hydrophobic interactions with residues Val170, Ile208, Ile236, and Tyr258. It induces a big shift in the position of Tyr258, forming a π–π interaction and closing pocket A at its extremity.

Montelukast is stabilized through hydrophobic interactions with residues Ile186, Leu189, Leu207, Ile236, and Tyr258. This is the only compound that induces a significative shift in the position of Ile186, closing pocket A, but leaving one of the aliphatic arms of montelukast outside.

## 3. Materials and Methods

### 3.1. Structure Identification and Analysis

The Protein Databank [45] and the Biofilms Structural Database [46] were explored to find molecular structures of *P. aeruginosa* PqsR/MvfR. A total of 12 X-ray structures were identified. Details are summarized in Table 5.

Structures 4JVC, 4JVD, and 4JVI (2013) [20] were the first to present the MvfR ligand binding domain in the apo form and in complex with its native agonist 2-nonyl-4-hydroxyquinoline or NNQ (4JVC and 4JVD, respectively). The authors have shown that the structure of this binding pocket is unusually large, in which a native AQ agonist is stabilized entirely by hydrophobic interactions [20]. They also presented a structure with an analogous to NNQ in the binding pocket (4JVI), i.e., the compound 3-amino-7-chloro-2-nonylquinazolin-4-one (QZN).

The PDB structure 6B8A (2018) presents the X-ray structure of the MvfR ligand-binding domain (LBD) in complex with one potent benzamide–benzimidazole (BB) inhibitor, i.e., 2-[(5-nitro-1*H*-benzimidazol-2-yl)sulfanyl]-*N*-(4-phenoxyphenyl)acetamide (or M64) [23]. The authors were able to demonstrate that this is a competitive inhibitor that binds in the same hydrophobic MvfR pocket as the natural inducers. The subtle conformational difference in the first three (4JVC, 4JVD, 4JVI) and fourth (6B8A) structures is mainly due to rearrangements in residues 181 to 191, in the presence of the natural inducers versus the inhibitor M64. This inhibitor, combined with sub-therapeutic doses of ciprofloxacin, was the first to show in vivo activity when tested in the treatment of a mouse lung infection model [28].

6Q7U, 6Q7V, and 6Q7W are structures of the LBD of MvfR in complex with 2-heptyl-4-quinolone or HHQ (6Q7U) and 2-aminopyridine derivatives, N-(4-(4-fluorophenyl)methyl)-6-(trifluoromethyl)pyridine-2,4-diamine or compound 11 (6Q7V), and N-(4-[3-(4-fluorophenyl)propyl]-6-(trifluoromethyl)pyridine-2,4-diamine or compound 20 (6Q7W). These derivatives were described by the Hartmann group as novel lead-like structure for the design of MvfR antagonists [22].

6TPR presented the MvfR LBD structure bound to another possible antagonist found through the optimization of a bacterial cell-based reporter assay hit. Soukarieh and co-workers were able to confirm that 2-[(5-methyl-[1,2,4]triazino[5,6-b]indol-3-yl)sulfanyl]-N-(4-pyridin-2-yloxyphenyl)ethanamide (or NV5) did reduce the biosynthesis of the alkylquinolone AIs even though it was unable to potentiate the effect of ciprofloxacin when combined [47].

6Z07, 6Z17, 6Z5K, and 6YZ3 are structures of the LBD of MvfR bound to four different thiazole-containing quinazolinones capable of inhibiting it. Grossman and his team were able to understand that the four ligands occupied the binding pocket in a similar manner to the AIs and that increasing the length of the aliphatic chain improved potency. For this reason, ligands 6-chloro-3((2-pentylthiazol-4-yl)methyl)quinazolin-4(3*H*)-one (or QAE) bound to 6Z5K and 6-chloro-3((2-hexylthiazol-4-yl)methyl)quinazolin-4(3*H*)-one (or Q25) bound to 6YZ3 were the ones that attenuated the production of the MvfR-regulated virulence factor pyocyanin while also showing low cytotoxicity in the in vitro assays [48].

**Figure 9:**
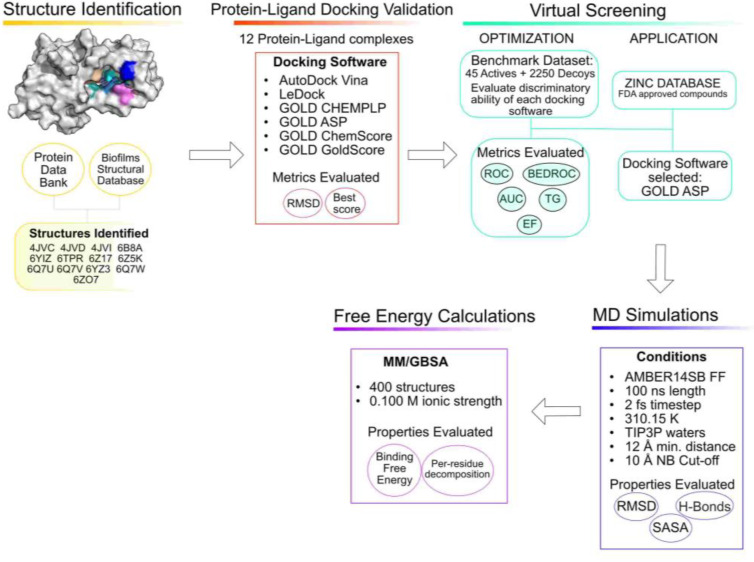
Workflow of the current study.

A schematic representation of the workflow used is presented in Figure 9.

Initially, all the PDB structures of the proteins were analyzed, aligned, and treated using Pymol 2.3.0. Crystallographic waters were removed, and ligands were extracted and saved in separate files. All the structures were aligned so that the docking coordinates and conditions were the same for each structure in the re-docking and cross-docking experiments. After that, Gasteiger charges and polar hydrogens were assigned to the proteins using AutoDockTools [49].

**Figure 10:**
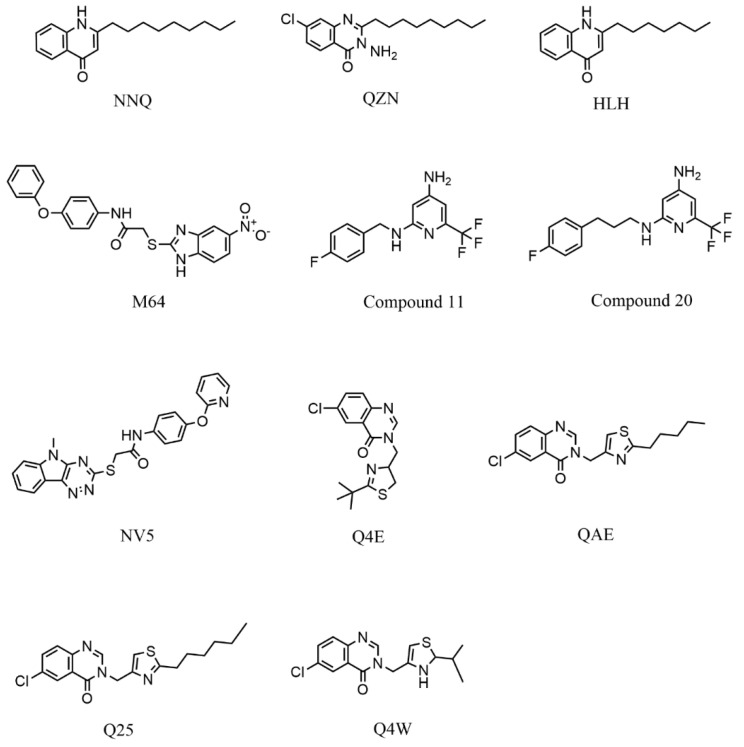
Chemical structures of the six crystallographic ligands.

The OpenBabel software [50] was used to prepare the ligands for docking. The chemical structures of the crystallographic ligands used in the validation stage are listed in Figure 10.

### 3.2. Protein–Ligand Docking Protocol Validation

Three docking software alternatives and six scoring functions (SFs) have been used for this study: Autodock Vina [51], LeDock, and Gold [52] (CHEMPLP, GoldScore, ChemScore, and ASP). The goal in testing all these different SFs is to find out which one works best for this specific target. The characteristics of the binding pocket and ligands in study have a big impact on the docking results [53,54].

To ensure reproducibility, the docking conditions were kept consistent for every software and every target. The parameters adjusted were the size of the docking box, the binding site definition, the number of runs, and search efficiency.

To validate the protocol, re-docking was performed by removing the crystallographic ligands and docking them again. This allows the user to evaluate the ability of the docking software to reproduce the geometry and orientation of the crystallographic pose. This was evaluated through the calculation of the root mean square deviation (RMSD) between the heavy atoms of the crystallographic and docked poses. Cross docking was also performed as a measure of validation. The goal here is to evaluate the robustness of different target structures and how they can accommodate different ligands. For cross docking, all the X-ray ligand structures isolated from the twelve PDB structures of this target were “docked” into the different X-ray structures. The RMSDs in both cases were calculated using DockRMSD [55].

The scoring functions used employ different metrics and scales. A good result is the one that presents a good score (depending on the SF used as different SFs use different metrics, it can be a high positive score—GOLD CHEMPLP, ASP, ChemScore, and GoldScore, or a more negative score—Vina, LeDock) and a RMSD below 2 Å.

### 3.3. Virtual Screening Protocol Optimization

The virtual screening protocol must be validated with benchmark datasets to ensure that it provides reliable results in the subsequent VS stage. This active versus decoy protocol is essential to validate the virtual screening conditions and evaluate the ability of each scoring function to discriminate between real binders and non-binders. A perfect scoring function would be capable of finding all the true binders early on and rank them higher than the decoys; however, that is not always the case due to the simplifications of the scoring functions. Virtual screening is meant to be fast as we want to screen large databases of compounds and that comes with a cost in accuracy [56]. So, special care was dedicated to improving the protocol’s ability in maximizing the number of known binders among the top solutions. For that, a PqsR/MvfR-specific virtual screening training library was prepared to evaluate and optimize the ability of the protocol in discriminating between known binders and non-binders.

After an initial query in the ChEMBL [57] and BindingDB [58] databases, 29 ligands were found to have reported experimental activity against MvfR. These 29 MVfR antagonists were first described by the Hartmann group in 2012 and 2013 using a ligand-based drug design approach [29] and fragment identification approach using surface plasmon resonance screening, respectively [24]. After going through the literature, an additional list of 11 manually curated active ligands was created [30,59,60,61]. Only the ligands that presented activity toward PqsR/MvfR were selected (i.e., compounds with IC50 values), raising the total of active molecules in the test set to 40.

Using the DUD-E [62] decoy generator, based on the ligands previously mentioned, a set of 50 decoys for each ligand was created. Decoys are molecules that resemble the ligands in their physical properties but are chemically and topologically different so that they are likely non-binders. This approach is useful to validate our scoring functions and build a benchmarking dataset. The total number of decoys generated was 2250, as 5 of the active molecules are present in two different protonated forms. As previously mentioned, both active molecules and decoys were prepared for docking using OpenBabel [50] and converted into pdbqt or mol2, depending on the specific docking software considered. A database of 2295 compounds was finally created and made ready for docking.

The six scoring functions were evaluated, and the metrics were calculated using a web-based application, Screening Explorer [63], as well as Excel. The metrics used for the evaluation of the VS results were the enrichment factor at 1%, receiver-operating characteristic (ROC) curves and the respective area under the curve (AUC), Boltzmann-enhanced discrimination of ROC (BEDROC), the robust initial enhancement (RIE), and total gain (TG). TG quantifies the discrimination of actives over decoys attributable to score variations. TG values over 0.25, combined with an AUC over 0.5, indicate a good performance and reproducibility from the VS protocol [63].

### 3.4. Virtual Screening for Drug Repurposing

After careful validation of the docking and virtual screening protocols, virtual screening (VS) experiments with the PqsR/MvfR target 6B8A were conducted using the ZINC FDA database of compounds to evaluate a possible drug repurposing strategy. For this stage, only the best scoring function and the X-ray structure that yield better discrimination of actives and decoys in the validation stages were selected.

The FDA-approved drugs library used was a subset of the ZINC [64] library, a free database of commercially available compounds for virtual screening. ZINC contains over 230 million purchasable compounds. At the time of the VS experiments, the FDA-approved drugs dataset had 3207 compounds that were all docked against the target.

The top 15 compounds identified in the VS were selected to be further studied by molecular dynamics simulations and free-energy calculations.

### 3.5. Molecular Dynamics Simulations

Molecular dynamic simulations are useful to validate the docking and VS predictions and provide insight into the protein–ligand interactions. It allows the study of the physical evolution of the system through time and is a valuable tool in the interpolation between theory and experiments [65,66,67]. The fifteen MvfR–ligand complexes were treated with the Leap module of Amber18 [68]. The MvfR protein was treated with the ff14SB force field [69]. All the FDA compounds were parameterized with Gaussian16 [70] using ANTECHAMBER, with RESP HF/6-31G(d) charges calculated with Gaussian16 and the general AMBER force field (GAFF) [71]. As control, two of the crystallographic ligands were used, i.e., NNQ as the natural agonist and M64 as an antagonist.

Sodium counter-ions were added to neutralize the overall charge in the system. The protein–ligand complexes were embedded into a box of TIP3P water molecules, whose edges were placed at least 12 Å away from each complex atom. Periodic boundaries were applied, and the long-range electrostatic interactions were calculated using the particle mesh Ewald summation method. The cut-off value for the short-range electrostatic and Lennard–Jones interactions was set at 10.0 Å. The hydrogen bonds were constrained using the SHAKE algorithm. A time step of 2 fs was used.

Four minimization steps were performed to remove clashed and applied in the following order: one water molecule (2500 steps); two hydrogens atoms (2500 steps); three side chains of all the amino acid residues (2500 steps); and four full systems (10,000 steps). After minimization, a molecular dynamics equilibration procedure was applied and divided into two stages: in the first stage (50 ps), the systems were gradually heated to 298 K using a Langevin thermostat at constant volume (NVT ensemble); in the second stage (50 ps), the density of the systems was further equilibrated at 298 K. Finally, the production phase was run for a total of 100 ns in an NPT ensemble at a pressure of 1 bar and a temperature of 298 K. This overall procedure has been previously used with success in the treatment of several biomolecular systems [72,73,74,75].

The molecular dynamic (MD) trajectories were analyzed using VMD [76] and the cpptraj tool [77].

### 3.6. Free-Energy Calculations

To estimate the binding free energies of the ligands toward MvfR, the molecular mechanics—generalized born surface area (MM-GBSA) method [78] was applied using the MM/PBSA.py script available in AMBER [79]. The calculations considered the last 90 ns of the MD simulation of every complex, using an interval of 10 frames, representing a total of 1800 frames per complex. For the MM/GBSA calculations, a salt concentration of 0.100 mol dm^−3^ was used.

The free-energy decomposition option was used to obtain information about the local interactions of the complex. Using per-residue decomposition, the contribution of each residue to the total free energy was estimated. This approach has been used with success in the study of several other systems, including quorum sensing inhibitors [72,73,80,81,82].

For the MvfR–FDA complexes with the top five binding free energies, two additional MD simulation replicas were performed per complex. MM-GBSA binding free energies were calculated, together with the additional MD simulations properties (average RMSd, SASA, number of hydrogen bonds), confirming the overall tendencies obtained (Suppelmentary Table S2).

## 4. Conclusions

A docking protocol was optimized for *P. aeruginosa* MfvR using the crystallographic ligands as validation tools in the reproducibility of the pose generated by the docking software. The VS protocol was adjusted based on known MfvR active ligands to obtain the best discriminatory ability between real binders and non-binders, and it was applied to a database of 3207 FDA-approved compounds.

The results obtained using the optimized VS protocol were further analyzed by MD simulations followed by free-energy calculations using the MM/GBSA method. This resulted in confirming five FDA-approved compounds with a high probability of exhibiting activity as *P. aeruginosa* QS inhibitors: venetoclax, indocyanine green, nilotinib, cabozantinib, and montelukast. Throughout the MD simulations, it was possible to observe shifts in the position of two residues that might be key in the activation mechanism of MvfR, i.e., Ile186 and Tyr258. π–π interactions with Tyr258 seem to have a crucial role in protein ligand affinity. In addition to the identification of this new potential application for drug repurposing of these molecules and demonstrating their mode of interaction, the protocol here described and validated can now be applied to large libraries of drug-like compounds to highlight new promising candidates for *P. aeruginosa* QS inhibitors. This study also highlighted the key role in molecular recognition played by residues Ile186 and Tyr258, a feature that can be taken into consideration in future rational drug design and optimization efforts targeting this important QS protein to develop new anti-biofilm molecules.

## Figures and Tables

**Table 1:** RMSD of the re-docking for the other targets and their respective crystallographic ligand.

Redocking RMSD (Å)								
PDB Code	Ligand	Vina	LeDock	CHEMPLP	GoldScore	ChemScore	ASP	Average per Target
4JVD	NNQ	6.67	3.51	2.16	3.20	1.18	2.68	3.23
4JVI	QZN	1.59	3.07	1.33	2.93	3.18	1.85	2.33
6B8A	M64	0.34	0.58	0.46	0.63	3.48	1.92	1.24
6Q7U	HLH	7.26	5.80	3.71	3.14	2.64	2.11	4.11
6Q7V	C11	5.77	6.16	3.49	5.73	3.76	1.77	4.98
6Q7W	C20	3.54	4.50	1.99	5.15	3.17	1.71	3.34
6TPR	NV5	9.15	5.23	1.54	1.16	4.59	1.35	3.84
6Z07	Q4E	1.22	1.41	0.92	0.87	1.31	1.32	1.18
6Z17	Q4W	1.18	7.25	1.59	4.08	2.32	1.96	3.06
6Z5K	QAE	1.43	4.46	1.17	8.13	3.95	1.60	3.46
6YZ3	Q25	1.11	7.67	1.25	0.99	3.46	1.56	2.67
Average by SF		3.57	4.51	1.78	3.27	3.00	1.81	

**Table 2:** Available X-ray structures of MvfR on the PDB and BSD.

	4JVI					6B8A				
	EF 1%	AUC%	TG	RIE	BEDROC	EF 1%	AUC	TG	RIE	BEDROC
Vina	10.23	0.68	0.21	2.34	0.14	10.35	0.63	0.19	2.13	0.13
CHEMPLP	10.40	0.55	0.08	2.22	0.13	5.20	0.54	0.07	2.07	0.12
ChemScore	5.20	0.49	0.005	1.68	0.10	2.60	0.52	0.02	1.65	0.10
ASP	10.40	0.66	0.21	2.39	0.14	10.39	0.66	0.25	2.33	0.14

**Table 3:** Top 15 hits of the FDA-approved drugs database.

Drug Name & Code	Description	Structure	ASP Score
Nilotinib	Bcr-Abl tyrosine kinase inhibitor (TKI) used in the treatment of chronic myelogenous leukemia (CML)	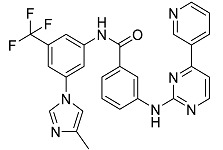	54.96
Indocyanine Green	Dye used in medical diagnosis. It has been used to measure cardiac output, liver function, and in ophthalmic angiography [33].	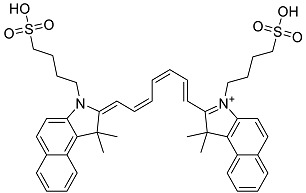	50.55
Lomitapide	Used to treat patients with Homozygous familial hypercholesterolaemia (HoFH). It is an inhibitor of MTP, an enzyme responsible for the synthesis of low-density lipoproteins in the liver [34].	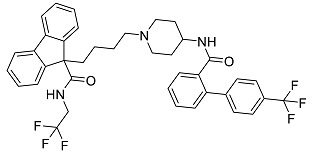	50.01
Valrubicin	Chemotherapy drug used to treat carcinoma in situ bladder tumors.	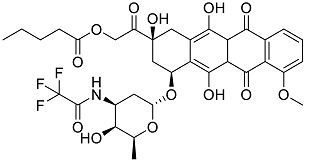	49.89
Lapatinib	Inhibitor of tyrosine kinase domains of epidermal growth factor receptor and human epidermal growth factor receptor (HER)-2. Used to treat metastatic HER-2 + breast cancer [35].	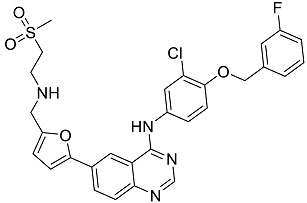	49.84
Pazopanib	Multitarget tyrosine kinase inhibitor approved for the treatment of multiple histological subtypes of soft tissue sarcoma (STS) [36].	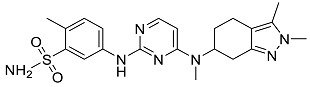	49.69
Ravicti	Used for the treatment of patients with urea cycle disorders (UCDs) [37].	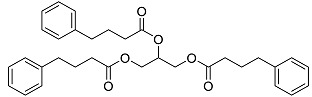	48.49
Cabozantinib	Tyrosine kinase inhibitor that targets pathways that have been linked to tumor growth. Used for the treatment of metastatic renal cell carcinoma [38].	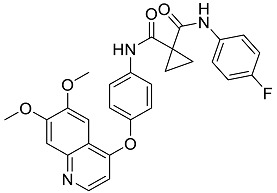	48.32
Venetoclax	Inhibitor of B-cell leukemia/lymphoma-2 protein [39].	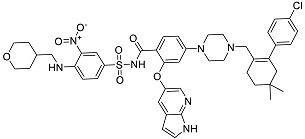	48.05
Isavuconazonium	Prodrug used as antifungal for the treatment of invasive aspergillosis and invasive mucormycosis [40].	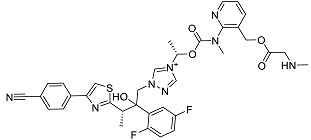	48.05
Emend	NK1 antagonist to prevent chemotherapy-induced nausea and vomiting.	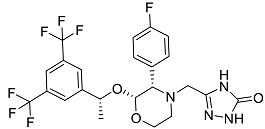	46.77
Amethopterin or Methotrexate	Analog and antagonist of folic acid, is commonly used in the treatment of a wide range of malignant and non-malignant diseases [41].	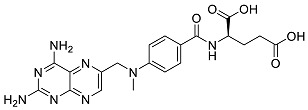	46.10
Cefsulodin	Broad-spectrum beta-lactamase stable cephalosporin with excellent activity against gram-negative bacilli, including *P. aeruginosa* [42].	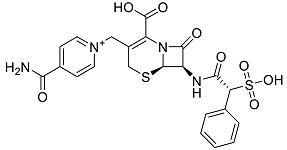	46.01
Montelukast	Leukotriene receptor antagonist (LTRAs) used for asthma treatment [43].	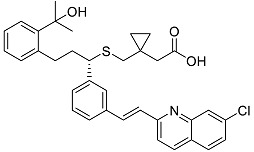	45.27
Cefoperazone	Is a parenteral, third-generation cephalosporin that can be given intravenously or intramuscularly [44].	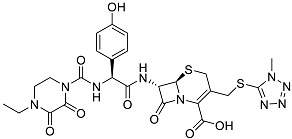	45.05

**Table 4:** Average protein RMSD values (Å), average ligand RMSD (Å), average MvfR–ligand complex SASA (Å^2^), percentage of SASA for the buried ligand, and average number of ligand hydrogen bonds, obtained from the MD simulations of MvfR–FDA complexes. ΔG binding energy determined using MM/GBSA and per-residue decomposition, calculated for the last 90 ns of the simulation. Compounds are ordered by increasing ΔGbind (kcal/mol).

ID	Average Protein RMSd (Å)	Average Ligand RMSD	SASA (Å^2^)	Percentage of Potential Ligand SASA Buried (%)	Average Number H Bonds	ΔGbind (kcal/mol)	Main Contributors (kcal/mol)
Venetoclax	2.3 ± 0.2	2.7 ± 0.4	344.8 ± 27.0	70.4 ± 0.02	0.5 ± 0.6	−70.1 ± 0.3	TYR258 (−4.9), ILE186 (−4.3), ILE236 (−2.9)
Indocyanine Green	2.2 ± 0.2	2.3 ± 0.2	298.9 ± 28.3	72.3 ± 0.03	1.1 ± 0.8	−58.6 ± 0.3	ILE186 (−4.2), ILE236 (−3.1), TYR258 (−3.1)
Nilotinib	2.0 ± 0.2	1.7 ± 0.4	105.2 ± 23.2	86.8 ± 0.03	0.1 ± 0.3	−48.1 ± 0.2	ILE186 (−2.4), LEU208 (−2.1), ILE236 (−3.4)
Cabozantinib	2.3 ± 0.3	1.6 ± 0.4	135.3 ± 40.5	82.3 ± 0.1	0.03 ± 0.2	−44.6 ± 0.2	ILE236 (−3.2), ILE186 (−2.4), LEU208 (−2.3)
Montelukast	2.2 ± 0.2	2.2 ± 0.4	228.8 ± 35.6	73.8 ± 0.04	0.04 ± 0.2	−43.2 ± 0.2	ILE236 (−2.8), LEU207 (−2.4), ILE263 (−2.0)
Cefoperazone	2.6 ± 0,3	2.9 ± 1.2	289.5 ± 41.4	66.7 ± 0.04	1.0 ± 0.9	−42.4 ± 0.4	ARG209 (−3.2), ILE236 (−2.5), LEU208 (−2.3)
Valrubicin	2.4 ± 0.4	3.3 ± 0.3	276.7 ± 31.4	70.6 ± 0.03	0.04 ± 0.2	−41.1 ± 0.2	LEU207 (−4.2), ILE236 (−2.9), TYR258 (−3.9)
Lomitapide	2.3 ± 0.3	3.8 ± 0.4	323.4 ± 81.6	65.6 ± 0.1	0.1 ± 0.3	−40.6 ± 0.3	ILE186 (−2.3), LEU208 (−1.9), ILE236 (−2.5)
M64 (antagonist)	2.2 ± 0.2	1.2 ± 0.2	90.4 ± 19.3	86.3 ± 0.03	0.1 ± 0.2	−39.0 ± 0.1	IlE236 (−3.2), ILE186 (−1.7), TYR258 (−1.7)
Emend	2.3 ± 0.4	1.5 ± 0.2	130.2 ± 25.8	80.7 ± 0.04	0.4 ± 0.5	−38.9 ± 0.2	ILE236 (−3.5), LEU207 (−109), TYR258 (−1.7)
Pazopanib	2.3 ± 0.2	1.9 ± 0.5	203.6 ± 40.5	69.4 ± 0.1	0.7 ± 0.8	−37.7 ± 0.3	LEU208 (−3.7), ILE236 (−3.1), SER196 (−2.0)
lapatinib	2.5 ± 0.4	2.8 ± 0.9	249.0 ± 56.7	69.6 ± 0.1	0.5 ± 0.7	−35.4 ± 0.3	LEU207 (−2.1), ILE236 (−2.9), ILE263 (−1.8)
Ravicti	2.1 ± 0.2	3.3 ± 0.7	263.8 ± 50.0	69.3 ± 0.1	0.03 ± 0.2	−34.5 ± 0.3	LEU207 (−2.6), ILE236 (−2.6), ILE263 (−1.7)
Cefsulodin	2.3 ± 0.2	1.6 ± 0.4	235.4 ± 39.9	66.5 ± 0.1	0.9 ± 0.8	−27.4 ± 0.3	LEU207 (−3.5), ILE236 (−2.2), LEU208 (−2.1)
NNQ (natural inducer)	2.3 ± 0.3	1.6 ± 0.4	154.8 ± 53.7	71.6 ± 0.1	0.1 ± 0.3	−26.1 ± 0.3	LEU208 (−1.7), ILE236 (−1.5)
Isavuconazonium	2.4 ± 0.2	2.7 ± 0.5	522.5 ± 47.4	42.7 ± 0.1	0.04 ± 0.2	−25.0 ± 0.1	TYR258 (−3.4), ILE186 (−2.6), LEU189 (−1.1)
Methotrexate	2.8 ± 0.4	2.4 ± 0.4	312.6 ± 69.2	53.8 ± 0.1	0.7 ± 0.8	−22.8 ± 0.3	ILE186 (−4.1), TYR258 (−3.4), ARG209 (−2.7)

**Table 5:** Available X-ray structures of MvfR on the PDB and BSD.

PDB Code	Protein	Resolution (Å)	Ligand	Strain	References
4JVC	Ligand-Binding Domain	2.50		UCBPP-PA14	[20]
4JVD	Ligand-Binding Domain	2.95	NNQ		
4JVI	Ligand-Binding Domain	2.90	QZN		
6B8A	Ligand-Binding Domain	2.65	M64	PAO1	[23]
6Q7U	Ligand-Binding Domain	3.15	HLH	PAO1	[22]
6Q7V	Ligand-Binding Domain	2.56	HLK		
6Q7W	Ligand-Binding Domain	2.82	HLQ		
6TPR	Ligand-Binding Domain	3.20	NV5	UCBPP-PA14	[47]
6Z07	Ligand-Binding Domain	2.95	Q4E	UCBPP-PA14	[48]
6Z17	Ligand-Binding Domain	3.15	Q4W		
6Z5K	Ligand-Binding Domain	3.20	QAE		
6YZ3	Ligand-Binding Domain	3.00	Q25		

## Data Availability

The database of compounds used in this project is available at https://zinc.docking.org/substances/home/ (accessed on 1 January 2021).

## Supplementary Materials

The following are available online at https://www.mdpi.com/article/10.3390/antibiotics11020185/s1, Table S1: Cross-docking scores obtained with the six scoring functions tested. Table S2. Summary of the MD simulations results on the replicas performed for the top 5 MvfR–FDA complexes.
