# Supplementary material for: Drug Repurposing Targeting Pseudomonas aeruginosa MvfR Using Docking, Virtual Screening, Molecular Dynamics, and Free-Energy Calculations
Source: Antibiotics (Basel). 2022 Jan 31;11(2):185. doi: 10.3390/antibiotics11020185 (PMC8868191; doi:10.3390/antibiotics11020185)
Supplement: Supplementary file 1 [file antibiotics-11-00185-s001.zip › antibiotics-1531837-supplementary.pdf]

# Drug repurposing in the battle against *P. aeruginosa* MvfR using Docking, Virtual Screening, Molecular Dynamics, and Free Energy Calculations

Tatiana F. Vieira[1,2], Rita P. Magalhães[1,2], Manuel Simões[3], Sérgio F. Sousa[1,2]

[1] UCIBIO/REQUIMTE, BioSIM – Departamento de Medicina, Faculdade de Medicina da Universidade Do Porto, Alameda Prof. Hernâni Monteiro, 4200-319 Porto, Portugal
[2] Associate Laboratory i4HB - Institute for Health and Bioeconomy, Faculdade de Medicina, Universidade do Porto, 4200-319 Porto, Portugal
[3] LEPABE Laboratory for Process Engineering, Environment, Biotechnology and Energy, Faculty of Engineering, University of Porto, Rua Dr. Roberto Frias, s/n, 4200-465 Porto, Portugal

**Table S1**. Cross-docking scores obtained with the six scoring functions tested**.**

| | | | | | | | | | | | | | |
|---|---|---|---|---|---|---|---|---|---|---|---|---|---|
| **Vina** | | | | | | | | | | | | | |
| | | **4JVC** | **4JVD** | **4JVI** | **6B8A** | **6Q7U** | **6Q7V** | **6Q7W** | **6TPR** | **6Z07** | **6Z17** | **6Z5K** | **6YZ3** |
| **4JVD** | **NNQ** | -7.20 | -7.20 | -7.10 | -7.30 | -7.70 | -7.30 | -7.50 | -7.70 | -7.50 | -7.50 | -7.50 | -7.60 |
| **4JVI** | **QZN** | -7.50 | -9.80 | -7.80 | -7.90 | -9.90 | -7.80 | -8.00 | -7.60 | -7.30 | -7.50 | -7.60 | -7.80 |
| **6B8A** | **M64** | -9.00 | -9.10 | -9.00 | -9.90 | -9.40 | -8.70 | -9.20 | -9.30 | -8.10 | -7.80 | -7.80 | -8.10 |
| **6Q7U** | **HLH** | -6.70 | -7.00 | -7.20 | -7.00 | -8.00 | -7.00 | -7.30 | -7.60 | -7.70 | -7.80 | -7.40 | -8.10 |
| **6Q7V** | **HLK** | -8.10 | -8.20 | -7.90 | -8.50 | -8.90 | -8.60 | -8.00 | -8.30 | -7.50 | -7.50 | -7.90 | -7.60 |
| **6Q7W** | **HLQ** | -7.60 | -7.60 | -8.10 | -8.10 | -8.60 | -7.60 | -7.50 | -8.10 | -7.20 | -7.30 | -7.60 | -7.40 |
| **6TPR** | **NV5** | -9.20 | -9.30 | -9.70 | -10.40 | -9.90 | -8.60 | -9.40 | -9.80 | -7.10 | -7.20 | -7.60 | -7.90 |
| **6Z07** | **Q4E** | -4.30 | -7.50 | -7.70 | -9.50 | -7.20 | -8.60 | -7.80 | -9.50 | -7.60 | -7.70 | -7.90 | -8.20 |
| **6Z17** | **Q4W** | -4.40 | -7.40 | -7.90 | -8.60 | -6.70 | -7.90 | -7.60 | -10.00 | -7.50 | -7.50 | -7.90 | -8.20 |
| **6Z5K** | **QAE** | -4.80 | -7.00 | -7.50 | -9.60 | -7.10 | -8.50 | -7.70 | -9.20 | -7.20 | -7.20 | -7.40 | -7.80 |
| **6YZ3** | **Q25** | -4.50 | -7.50 | -8.00 | -9.70 | -7.20 | -8.00 | -7.70 | -9.80 | -7.20 | -7.60 | -7.60 | -8.00 |
| **LeDock** | | | | | | | | | | | | | |
| | | **4JVC** | **4JVD** | **4JVI** | **6B8A** | **6Q7U** | **6Q7V** | **6Q7W** | **6TPR** | **6Z07** | **6Z17** | **6Z5K** | **6YZ3** |
| **4JVD** | **NNQ** | -5.08 | -5.06 | -5.15 | -5.49 | -5.32 | -4.91 | -5.23 | -5.11 | -5.64 | -5.83 | -6.04 | -6.13 |
| **4JVI** | **QZN** | -6.27 | -6.19 | -6.44 | -6.92 | -6.25 | -5.97 | -6.75 | -5.66 | -5.81 | -5.97 | -6.68 | -6.76 |

| | | | | | | | | | | | | | |
|---|---|---|---|---|---|---|---|---|---|---|---|---|---|
| **6B8A** | **M64** | -8.40 | -6.90 | -7.26 | -7.87 | -8.30 | -8.07 | -7.36 | -7.80 | -5.97 | -6.44 | -6.91 | -6.97 |
| **6Q7U** | **HLH** | -4.76 | -4.77 | -4.95 | -5.24 | -4.98 | -4.73 | -5.15 | -4.98 | -5.31 | -5.94 | -5.97 | -6.03 |
| **6Q7V** | **HLK** | -5.69 | -6.37 | -5.78 | -6.23 | -6.15 | -5.88 | -6.23 | -5.97 | -5.42 | -6.05 | -6.30 | -6.27 |
| **6Q7W** | **HLQ** | -6.19 | -6.17 | -6.29 | -6.44 | -6.59 | -6.14 | -6.04 | -5.89 | -5.22 | -5.44 | -5.95 | -6.12 |
| **6TPR** | **NV5** | -6.98 | -7.70 | -6.39 | -7.64 | -7.38 | -7.20 | -8.03 | -7.18 | -5.05 | -5.66 | -6.10 | -6.23 |
| **6Z07** | **Q4E** | -3.31 | -4.78 | -6.01 | -7.64 | -4.58 | -6.58 | -6.02 | -6.45 | -5.40 | -5.63 | -6.14 | -6.05 |
| **6Z17** | **Q4W** | -3.32 | -5.23 | -6.33 | -8.38 | -4.74 | -6.04 | -6.67 | -6.96 | -5.75 | -6.00 | -6.58 | -6.26 |
| **6Z5K** | **QAE** | -3.30 | -5.29 | -6.40 | -7.60 | -4.8 | -5.73 | -6.16 | -6.89 | -5.39 | -5.77 | -6.25 | -6.09 |
| **6YZ3** | **Q25** | -3.28 | -5.18 | -6.56 | -7.58 | -4.77 | -5.74 | -5.94 | -6.70 | -5.33 | -5.56 | -6.13 | -6.07 |
| **CHEMPLP** | | | | | | | | | | | | | |
| | | **4JVC** | **4JVD** | **4JVI** | **6B8A** | **6Q7U** | **6Q7V** | **6Q7W** | **6TPR** | **6Z07** | **6Z17** | **6Z5K** | **6YZ3** |
| **4JVD** | **NNQ** | 62.09 | 61.31 | 63.41 | 67.70 | 65.77 | 66.06 | 67.95 | 67.98 | 53.17 | 58.96 | 68.64 | 72.91 |
| **4JVI** | **QZN** | 71.07 | 68.84 | 67.23 | 75.11 | 72.53 | 69.56 | 74.36 | 67.83 | 58.28 | 57.54 | 69.46 | 72.89 |
| **6B8A** | **M64** | 85.59 | 78.33 | 84.79 | 89.29 | 86.21 | 79.93 | 83.48 | 81.86 | 68.13 | 66.34 | 76.52 | 80.6 |
| **6Q7U** | **HLH** | 54.28 | 53.30 | 54.45 | 59.92 | 64.38 | 60.58 | 60.26 | 59.95 | 56.46 | 54.85 | 70.67 | 68.83 |
| **6Q7V** | **HLK** | 58.78 | 57.29 | 57.26 | 59.59 | 58.36 | 61.11 | 63.43 | 55.03 | 61.67 | 60.89 | 70.69 | 72.26 |
| **6Q7W** | **HLQ** | 67.57 | 66.06 | 65.66 | 69.42 | 67.04 | 63.14 | 69.53 | 63.51 | 57.50 | 60.05 | 71.86 | 74.33 |
| **6TPR** | **NV5** | 73.76 | 82.01 | 81.87 | 95.86 | 86.80 | 79.95 | 84.02 | 84.78 | 54.41 | 59.58 | 70.63 | 77.16 |
| **6Z07** | **Q4E** | 36.35 | 63.76 | 72.26 | 81.83 | 58.04 | 61.02 | 66.39 | 84.52 | 61.42 | 63.58 | 75.00 | 80.88 |
| **6Z17** | **Q4W** | 36.04 | 69.41 | 70.72 | 78.30 | 56.09 | 56.09 | 63.31 | 80.02 | 59.86 | 60.55 | 75.17 | 78.93 |
| **6Z5K** | **QAE** | 42.04 | 64.79 | 71.27 | 85.47 | 59.02 | 57.86 | 64.94 | 82.87 | 59.63 | 61.10 | 74.84 | 77.03 |
| **6YZ3** | **Q25** | 41.35 | 64.08 | 72.96 | 91.05 | 56.69 | 60.03 | 65.94 | 85.25 | 59.44 | 62.19 | 73.16 | 77.13 |
| **GoldScore** | | | | | | | | | | | | | |
| | | **4JVC** | **4JVD** | **4JVI** | **6B8A** | **6Q7U** | **6Q7V** | **6Q7W** | **6TPR** | **6Z07** | **6Z17** | **6Z5K** | **6YZ3** |
| **4JVD** | **NNQ** | 56.25 | 54.80 | 55.82 | 55.77 | 52.66 | 47.76 | 54.4 | 54.92 | 52.60 | 57.29 | 56.43 | 60.30 |
| **4JVI** | **QZN** | 56.75 | 61.98 | 62.01 | 64.44 | 52.14 | 51.71 | 58.95 | 53.00 | 51.04 | 51.49 | 58.48 | 60.53 |

| | | | | | | | | | | | | | |
|---|---|---|---|---|---|---|---|---|---|---|---|---|---|
| **6B8A** | **M64** | 73.57 | 69.82 | 72.26 | 76.77 | 68.40 | 70.76 | 70.04 | 71.56 | 56.73 | 53.93 | 61.64 | 64.94 |
| **6Q7U** | **HLH** | 49.42 | 46.56 | 51.66 | 50.59 | 47.06 | 48.62 | 51.15 | 49.83 | 58.39 | 63.61 | 54.59 | 59.26 |
| **6Q7V** | **HLK** | 50.25 | 51.81 | 49.37 | 54.93 | 48.17 | 49.27 | 49.56 | 45.58 | 53.51 | 51.64 | 59.25 | 58.64 |
| **6Q7W** | **HLQ** | 46.03 | 49.32 | 44.24 | 49.13 | 50.31 | 48.63 | 47.11 | 55.24 | 54.88 | 53.77 | 61.76 | 60.73 |
| **6TPR** | **NV5** | 70.97 | 65.11 | 68.86 | 79.12 | 65.91 | 74.45 | 70.11 | 75.15 | 51.76 | 60.15 | 57.56 | 57.14 |
| **6Z07** | **Q4E** | 30.07 | 54.87 | 58.81 | 73.51 | 52.89 | 52.89 | 51.19 | 73.58 | 53.88 | 56.44 | 59.80 | 59.98 |
| **6Z17** | **Q4W** | 28.74 | 54.99 | 59.96 | 73.43 | 53.37 | 49.71 | 51.38 | 73.69 | 57.26 | 55.68 | 62.71 | 65.23 |
| **6Z5K** | **QAE** | 31.29 | 54.09 | 57.26 | 73.50 | 57.35 | 48.69 | 52.16 | 71.04 | 54.14 | 59.88 | 65.56 | 61.80 |
| **6YZ3** | **Q25** | 31.66 | 53.9 | 58.95 | 73.63 | 58.10 | 50.78 | 52.37 | 73.24 | 51.61 | 49.26 | 60.29 | 59.16 |
| **ChemScore** | | | | | | | | | | | | | |
| | | **4JVC** | **4JVD** | **4JVI** | **6B8A** | **6Q7U** | **6Q7V** | **6Q7W** | **6TPR** | **6Z07** | **6Z17** | **6Z5K** | **6YZ3** |
| **4JVD** | **NNQ** | 33.67 | 33.54 | 34.44 | 34.66 | 30.05 | 33.80 | 36.57 | 33.97 | 24.18 | 27.72 | 33.92 | 34.75 |
| **4JVI** | **QZN** | 33.99 | 30.98 | 36.02 | 34.35 | 30.59 | 30.91 | 34.80 | 32.23 | 23.80 | 27.61 | 34.04 | 35.11 |
| **6B8A** | **M64** | 34.64 | 32.89 | 35.30 | 37.39 | 34.51 | 32.07 | 31.16 | 35.78 | 28.6 | 30.63 | 32.92 | 34.96 |
| **6Q7U** | **HLH** | 29.57 | 29.41 | 31.17 | 30.39 | 23.63 | 30.81 | 31.53 | 28.99 | 25.73 | 27.43 | 31.97 | 33.19 |
| **6Q7V** | **HLK** | 24.65 | 24.41 | 25.53 | 25.05 | 27.92 | 25.15 | 25.01 | 24.16 | 27.12 | 29.12 | 33.57 | 35.35 |
| **6Q7W** | **HLQ** | 27.58 | 28.04 | 28.56 | 29.00 | 25.37 | 27.32 | 27.65 | 28.22 | 26.18 | 30.07 | 32.54 | 33.83 |
| **6TPR** | **NV5** | 33.99 | 32.60 | 33.47 | 39.59 | 31.97 | 37.08 | 35.67 | 34.11 | 27.03 | 28.48 | 34.48 | 34.93 |
| **6Z07** | **Q4E** | 19.50 | 33.85 | 31.70 | 34.33 | 30.65 | 25.07 | 27.92 | 34.92 | 27.33 | 29.17 | 32.61 | 34.73 |
| **6Z17** | **Q4W** | 20.95 | 35.89 | 34.53 | 33.72 | 32.01 | 25.31 | 28.67 | 35.41 | 26.57 | 30.82 | 34.53 | 35.3 |
| **6Z5K** | **QAE** | 20.66 | 33.52 | 31.58 | 36.00 | 30.36 | 24.43 | 27.38 | 35.69 | 25.56 | 30.32 | 32.65 | 35.02 |
| **6YZ3** | **Q25** | 20.19 | 34.03 | 32.27 | 38.53 | 30.08 | 24.38 | 27.82 | 36.48 | 26.90 | 29.09 | 32.64 | 34.24 |
| **ASP** | | | | | | | | | | | | | |
| | | **4JVC** | **4JVD** | **4JVI** | **6B8A** | **6Q7U** | **6Q7V** | **6Q7W** | **6TPR** | **6Z07** | **6Z17** | **6Z5K** | **6YZ3** |
| **4JVD** | **NNQ** | 27.04 | 27.14 | 26.91 | 29.53 | 27.6 | 27.13 | 27.15 | 27.45 | 29.94 | 28.46 | 31.59 | 32.81 |

| | | | | | | | | | | | | | |
|---|---|---|---|---|---|---|---|---|---|---|---|---|---|
| **4JVI** | **QZN** | 30.50 | 30.58 | 33.77 | 33.12 | 31.33 | 29.84 | 29.97 | 31.21 | 29.84 | 28.09 | 31.89 | 33.85 |
| **6B8A** | **M64** | 41.90 | 40.71 | 41.85 | 47.36 | 46.78 | 39.24 | 38.05 | 40.65 | 30.92 | 30.51 | 33.43 | 34.95 |
| **6Q7U** | **HLH** | 24.30 | 23.72 | 24.83 | 26.19 | 29.67 | 24.07 | 23.78 | 24.98 | 30.56 | 29.10 | 32.40 | 33.46 |
| **6Q7V** | **HLK** | 27.09 | 27.85 | 28.40 | 28.50 | 24.37 | 28.7 | 28.24 | 26.47 | 29.77 | 29.44 | 31.88 | 33.34 |
| **6Q7W** | **HLQ** | 31.27 | 32.32 | 31.09 | 32.44 | 30.45 | 31.22 | 32.78 | 31.26 | 28.59 | 28.07 | 30.52 | 32.60 |
| **6TPR** | **NV5** | 32.54 | 34.13 | 37.49 | 35.94 | 38.22 | 33.15 | 33.42 | 36.23 | 30.45 | 28.59 | 32.46 | 33.47 |
| **6Z07** | **Q4E** | 11.47 | 27.48 | 31.00 | 40.65 | 24.87 | 29.32 | 32.23 | 34.43 | 30.43 | 29.19 | 32.96 | 33.93 |
| **6Z17** | **Q4W** | 11.51 | 27.55 | 30.49 | 41.03 | 24.84 | 27.37 | 31.22 | 33.07 | 31.89 | 30.01 | 33.33 | 33.95 |
| **6Z5K** | **QAE** | 11.42 | 27.63 | 31.43 | 43.13 | 24.77 | 28.34 | 31.96 | 35.80 | 30.02 | 29.08 | 33.11 | 33.41 |
| **6YZ3** | **Q25** | 11.48 | 27.69 | 31.57 | 42.74 | 25.40 | 28.92 | 32.12 | 35.23 | 29.73 | 28.86 | 32.88 | 34.22 |

**Table S2**. Summary of the MD simulations results on the replicas performed for the top 5 MvfR-FDA complexes. Average protein RMSD values (Å), average ligand RMSD (Å), Average MvfR-ligand complex SASA (Å2), Percentage of SASA for the buried ligand, average number of ligand hydrogen bonds.

| ID | Average Protein RMSd (Å) | Average Ligand RMSD | SASA (Å$^2$) | Percentage of Potential Ligand SASA Buried (%) | Average number H bonds | ΔGbind (kcal/mol) | Main Contributors (kcal/mol) |
|---|---|---|---|---|---|---|---|
| **Venetoclax** | 2.3 ± 0.2 | 2.7 ± 0.4 | 344.8 ± 27.0 | 70.4 ± 0.02 | 0.5 ± 0.6 | -70.1 ± 0.3 | TYR258 (-4.9), ILE186 (-4.3), ILE236 (-2.9) |
| | 2.7 ± 0.2 | 2.9 ± 0.4 | 307.8 ± 42.3 | 72.2 ± 0.04 | 0.4 ± 0.6 | -64.1 ± 0.3 | TYR258 (-3.6), ILE186 (-3.3), ILE236 (-2.5) |
| | 2.2 ± 0.2 | 2.9 ± 0.2 | 352.8 ± 33.2 | 68.5 ± 0.03 | 0.4 ± 0.5 | -66.2 ± 0.2 | TYR258 (-4.6), ILE186 (-3.2), ILE236 (-3.1) |
| **Indocyanine Green** | 2.2 ± 0.2 | 2.3 ± 0.2 | 298.9 ± 28.3 | 72.3 ± 0.03 | 1.1 ± 0.8 | -58.6 ± 0.3 | ILE186 (-4.2), ILE236 (-3.1), TYR258 (-3.1) |
| | 2.2 ± 0.2 | 2.4 ± 0.2 | 283.5 ± 32.0 | 73.6 ± 0.03 | 1.0 ± 0.7 | -59.2 ± 0.3 | ILE186 (-4.1), ILE236 (-3.3), TYR258 (-3.2) |
| | 2.6 ± 0.4 | 2.3 ± 0.3 | 279.4 ± 32.9 | 74.1 ± 0.03 | 0.8 ± 0.6 | -55.3 ± 0.3 | ILE186 (-3.3), ILE236 (-3.1), TYR258 (-3.0) |
| **Nilotinib** | 2.0 ± 0.2 | 1.7 ± 0.4 | 105.2 ± 23.2 | 86.8 ± 0.03 | 0.1 ± 0.3 | -48.1 ± 0.2 | ILE186 (-2.4), LEU208 (-2.1), ILE236 (-3.4) |
| | 2.2 ± 0.2 | 0.9 ± 0.1 | 71.6 ± 18.6 | 91.0 ± 0.02 | 0.3 ± 0.4 | -53.3 ± 0.2 | ILE186 (-2.7), LEU208 (-1.7), ILE236 (-3.8) |
| | 2.7 ± 0.5 | 1.0 ± 0.2 | 84.7 ± 23.2 | 89.3 ± 0.03 | 0.1 ± 0.3 | -50.7 ± 0.2 | ILE186 (-2.6), LEU208 (-1.5), ILE236 (-3.7) |
| **Cabozantinib** | 2.3 ± 0.3 | 1.6 ± 0.4 | 135.3 ± 40.5 | 82.3 ± 0.1 | 0.03 ± 0.2 | -44.6 ± 0.2 | ILE236 (-3.2), ILE186 (-2.4), LEU208 (-2.3) |
| | 2.3 ± 0.3 | 1.2 ± 0.2 | 147.4 ± 46.3 | 80.7 ± 0.1 | 0.3 ± 0.4 | -43.1 ± 0.2 | ILE236 (-3.2), ILE186 (-2.5), LEU208 (-2.4) |
| | 2.3 ± 0.2 | 1.4 ± 0.1 | 104.6 ± 25.0 | 86.4 ± 0.03 | 0.02 ± 0.1 | -47.9 ± 0.2 | ILE236 (-3.4), ILE186 (-1.4), LEU208 (-2.1) |
| **Montelukast** | 2.2 ± 0.2 | 2.2 ± 0.4 | 228.8 ± 35.6 | 73.8 ± 0.04 | 0.04 ± 0.2 | -43.2 ± 0.2 | ILE236 (-2.8), LEU207 (-2.4), ILE263 (-2.0) |
| | 2.2 ± 0.2 | 2.6 ± 0.4 | 231.9 ± 48.4 | 73.3 ± 0.1 | 0.2 ± 0.4 | -44.9 ± 0.3 | ILE236 (-3.1), LEU207 (-2.3), ILE263 (-2.2) |
| | 2.2 ± 0.2 | 2.5 ± 0.4 | 261.2 ± 38.4 | 70.2 ± 0.04 | 0.2 ± 0.4 | -41.0 ± 0.2 | ILE236 (-2.9), LEU207 (-2.7), ILE263 -2.0) |
